# Behavioral flexibility: A review, a model, and some exploratory tests

**DOI:** 10.3758/s13420-020-00421-w

**Published:** 2020-02-10

**Authors:** Stephen E. G. Lea, Pizza K. Y. Chow, Lisa A. Leaver, Ian P. L. McLaren

**Affiliations:** Department of Psychology, Washington Singer Laboratories, University of Exeter, Exeter, EX4 4QG UK

**Keywords:** Cognitive ethology, Comparative cognition, Operant conditioning, Acquisition

## Abstract

This paper aimed to explore and clarify the concept of behavioral flexibility. A selective literature review explored how the concept of behavioral flexibility has been used in ways that range from acknowledging the fact that animals’ behavior is not always bounded by instinctual constraints, to describing the variation between species in their capacity for innovative foraging, a capacity that has repeatedly been linked to having a brain larger than would be predicted from body size. This wide range of usages of a single term has led to some conceptual confusion. We sought to find a more precise meaning for behavioral flexibility by representing it within a simple formal model of problem solving. The key to our model is to distinguish between an animal’s state of knowledge about the world and its observable behavior, using a construct of response strength to represent that underlying knowledge. We modelled behavioral flexibility as a parameter in the function that transforms response strengths into observable response probabilities. We tested this model in simulations based on some recent experimental work on animal problem solving. Initial results showed that parametric manipulation can mimic some of the behavioral effects that have been attributed to flexibility.

## Introduction

The purpose of this paper was to explore the concept of behavioral flexibility. The term is widely used: down to the end of 2018, Web of Science records 1,867 publications that used it in their title, abstract, or keywords, with 167 such references in 2018 alone. While some of these publications are not relevant to comparative cognition, many are. Unsurprisingly, given the extent to which the term is used, authors are not consistent in what they mean by it. Accordingly, we do not start this paper with a definition of behavioral flexibility. Instead, we attempt to extract a core meaning for it within comparative cognition, by tracing the way it has been used historically, and by collating the consequences that have been associated, empirically, with greater or lesser degrees of behavioral flexibility. We are not offering an exhaustive review of the literature on behavioral flexibility, but rather attempting to identify its key points by means of selected examples; inevitably, these examples are biased towards areas of study, or study species, in which we have personal experience; in particular, we concentrate on examples involving animals extracting food from the environment. We then attempt to make the meaning of flexibility more precise by formulating a simple model of learning in problem-solving situations, in which flexibility is commonly supposed to play a part. In this model we introduce a parameter that aims to capture what we mean by flexibility. Thus, we hope to end with a definition, rather than starting with one. Simulations based on the model test the extent to which we have indeed captured the essence of behavioral flexibility; however, it is inevitable that any precise definition will be inconsistent with some of the historical uses of the concept.

## Historical development of the concept of behavioral flexibility within comparative cognition

To understand the origins of the concept of behavioral flexibility, we have to recall a conflict over the explanation of animal behavior that now seems quaint. In the 1930s–1950s there was a sharp distinction between two approaches. On the one hand, comparative psychologists (mainly Anglo-Saxon) were seeking to explain behavior in terms of the experiences of reward and punishment that an individual animal had had. On the other hand, ethologists (mainly European) were seeking to explain behavior in terms of the species to which an animal belonged, and the evolutionary and developmental history characteristic of that species. Of course, things were much more nuanced than this crude summary implies. But these polar positions illuminate the first significant use of the term “behavioral flexibility” that we have been able to find in the literature of what we would now call comparative cognition. Poirier (1969) introduces the term to cover the variations in behavior he observed between and within troops of Niligri langurs (*Semnopithecus johnii*) in the wild. In particular, troops varied in their diet, and although these variations were partly explained by variations in availability (in turn due to variations in the intrusion of human activities into the monkeys’ home ranges), Poirier found that a full explanation required an appeal to learned traditions of food acceptability. Within troops, younger animals and females were more ready to accept new foods. Thus behavior – even species-typical behavior – could not be accounted for by the kind of fixed-action pattern that the early ethologists described (e.g., Lorenz, 1932/1970; Tinbergen & Perdeck, 1950), whose very name implied inflexibility.

Fifty years after the publication of Poirier’s paper, all of this seems completely obvious, and we certainly no longer think in terms of a dichotomy between “innate” and “learned” behaviors. Poirier, however, found it necessary to devote most of the Discussion of his results to the point that “much of primate behavior results from a sizable learning component” and that “animals do not live by innate behavior alone” (p. 130). In this earliest, and still current, sense, therefore, “behavioral flexibility” simply encompasses all learning to adapt to the particular conditions of an individual’s environment – in effect, the whole of animal cognition.

### Behavior is not always flexible

To say that behavior is flexible in this sense can seem like a glimpse of the obvious. To make sense of it, we need to remember the extent to which the behavior of individual animals is often inflexible – or is thought to be. Animals do not behave at random, and if there is no reason to change their behavior, we should not expect that they will do so. Some examples of relative inflexibility include:

#### Individual foraging site fidelity

Many animals live, roost, or nest in colonies to which they return either after each day’s foraging or (especially when incubating mates or young are being fed) repeatedly during foraging. It is common for such colonial central place foragers to be faithful to a particular foraging site, even though they would be equally capable of reaching, and using, the sites used by other colony members; and even though these areas are not defended as a territory. E.A. Morgan, Hassall, Redfern, Bevan, and Hamer (2019) list a number of examples of such individual foraging site fidelity, and explore its extent – and limitations – in the European shag (*Phalacrocorax aristotelis*), a colonially nesting diving bird. Across the 70 shags they studied, the overlap between the utilization distributions of foraging sites on repeated trips varied considerably, from 0.06 to 0.75 on a scale from 0 to 1; but it was far higher than would be expected by chance. Furthermore, such inflexibility may be advantageous: female shags with a higher repeatability score tended to hatch their eggs earlier and were in better body conditions than those with lower scores. Obviously, since these preferred foraging sites consisted of a patch of open sea, there was no question of territorial defense.

#### Consistency of foraging method

Even when attacking a particular kind of prey in a given location, animals may have more than one method of attack available. In many coastal sites in northern Europe, Eurasian oystercatchers (*Haematopus ostralegus*) prey on the common mussel, *Mytilus edulis*, especially where large beds of mussels are found, as in the drowned estuaries of south-west England and Brittany. Three distinct techniques that oystercatchers use for opening mussels have been documented. One involves stabbing at the opening between the valves of the shell. The other two both involve hammering through the shell. However, in one case the bird hammers through the dorsal surface of the shell *in situ*, while in the other it removes the mussel to an “anvil,” a nearby location where it will be held in place while the oystercatcher strikes it, and hammers through the ventral surface of the shell (Goss-Custard, Durell, & Ens, 1982; Norton-Griffiths, 1967). Goss-Custard et al. observed 28 individually marked oystercatchers; roughly equal numbers used each technique, and all but one of the birds used a single technique exclusively. Another example of consistent variation in foraging method has been described in humpback whales: although humpbacks generally use bubbles to herd fish prey, sometimes in complex ways, the details (for example, whether or not bubble clouds are used) vary between individuals and regions (Wiley et al., 2011).

#### Consistency of foraging parameters

Within a particular foraging method, the parameters of the behavior may show consistent individual differences. For example, air-breathing divers face a trade-off between time available for foraging underwater and time available for replenishing oxygen stocks on the surface; in general, we would expect the balance to be struck at an underwater duration somewhat less than the maximum physiologically possible (Kramer, 1988). Potier, Carpentier, Grémillet, Leroy, and Lescroël (2015) examined the repeatability of the parameters of diving behavior in the great cormorant (*Phalacrocorax carbo*), and found considerable variation in repeatability between individuals and between parameters; in general, however, repeatability was substantially greater than would be expected by chance. For some parameters (e.g., mean time underwater per dive) high repeatability – inflexibility – was associated with reduced overall foraging efficiency, a likely proxy for fitness. But for others (e.g., mean time spent underwater per diving bout) the relation was positive.

None of these examples is absolute. We have already seen that there was individual variation in repeatability of behavior in both the diving examples; and in the case of oystercatcher foraging methods, subsequent work by Goss-Custard and colleagues showed that individuals tend to shift from stabbing to hammering as they grow older (Goss-Custard & Sutherland, 1984), and that at each age, the predominant method is the one associated with the higher fitness (Durell, Goss- Custard, Caldow, Malcolm, & Osborn, 2001). But they do show that flexibility is neither ubiquitous nor always advantageous. Indeed, Davis, Schapiro, Lambeth, Wood, and Whiten (2019) have argued that, because of overall limitations on cognitive capacity, some degree of behavioral inflexibility (or, as they term it, conservatism) is an inevitable result as individual behaviors become more complex. In a series of experiments on captive chimpanzees, they showed that there was little conservatism (i.e., high flexibility) when chimpanzees were required to inhibit a simple well-established response (using one of two available handles) to extract food from a puzzle box. But the apes showed greater conservatism when the apes had to inhibit a more complex, also well-established response, involving two successive doors and removing an obstacle in order to access food in a different puzzle box.

### Flexibility as variation in behavior in response to environmental change

It is, therefore, not unreasonable for ethologists to start from an assumption of behavioral inflexibility: that an animal’s behavior can be predicted from what kind of animal it is. Nevertheless, behavioral ecology has revealed many cases where the behavior of a given species varies depending on the environmental conditions, either between individuals living in different environments, or within the lifetime of an individual. Indeed, many of the optimizing models that have been the theoretical bedrock of behavioral ecology predict that such variation should occur. The following brief lists give a few examples of situations where it has been demonstrated. They are selected because their authors specifically referred to behavioral variation as a function of environmental change as “behavioral flexibility,” and they thus show the enduring use of this terminology over a two-decade period.

In some cases, “behavioral flexibility” is used to describe cases where behavior varies between populations living in different environments, or between groups within the population (e.g., between sexes or age classes). For example, Price, Tonn, and Paszkowski (1991) demonstrated variations in the prey choice, habitat use, and activity pattern between males and females and between adults and juveniles in fathead minnows (*Pimephales promelas*); Klett-Mingo, Pavoni, and Gil (2016) found that vigilance in great tits (*Parus major*) living near a major airport varies as a function of aircraft noise; and Ben Cohen and Dor (2018) found that the exploratory behavior and neophobia of house sparrows (*Passer domesticus*) varies along a climate gradient in the same way as morphological characteristics such as size and the darkness of the plumage.

In other cases, “behavioral flexibility” is used to describe behavior change that we can be confident is occurring within individuals, either because individuals have been tracked, or because the changes affect whole populations as a function of time of day or year. For example, Thompson and Baldassarre (1991) showed that the activity patterns of several species of migrant ducks in Yucatan change with foraging site and temperature; Knight, Vanjaarsveld, and Mills (1992) observed the unusual phenomenon of allo-suckling in spotted hyenas (*Crocuta crocuta*) after a prolonged drought; Palagi, Antonacci, and Cordoni (2007) showed how a play signal could switch the response to an ambiguous behavior from aggression to play in young lowland gorillas (*Gorilla gorilla gorilla*); and Christensen-Dalsgaard, May, and Lorentsen (2018) found that foraging site use in black-legged kittiwakes (*Rissa tridactyla*) varies as a function of distance from nest site, in interaction with weather conditions.

### Flexibility mediated by learning and culture in the wild

Where behavior differs between populations, the differences could be mediated by genetic, instinctual mechanisms, and indeed in some cases authors (e.g., Ben Cohen & Dor, 2018) have sought to correlate behavioral and genetic variation. Other authors, however, invoke the term “behavioral flexibility” to mark the fact that animals learn to adapt their behavior to their varying environments. Such learning inevitably builds on the characteristic behavioral repertoire of the species concerned, and several general theories have been produced to detail the interaction of evolutionary and cognitive processes in bringing about adaptive behavioral variation. Examples include the theories of Bindra (1978), West-Eberhard (2003, chapter 18), Mery and Burns (2010), and Fawcett, Hamblin, and Giraldeau (2013) for behavior in general, and Taborsky and Oliveira (2012) for social behavior in particular.

In looking for demonstrations of the role of learning in adaptive phenotypic variation under natural conditions, we face the difficulty of disentangling learning effects from those of genetic variation. Furthermore, the mere occurrence of learning is not sufficient, since learning is involved in the emergence of many behaviors that are typical of a species (Hailman, 1969). What we are looking for are cases where behavioral differences within a population (or within the lifetime of an individual) can be attributed to different learning experiences. Despite the difficulties, learning has been claimed as an explanation of natural behavioral flexibility across a wide taxonomic range, including in the feeding of gastropods (reviewed by Elliott & Susswein, 2002), in prey capture by ladybird beetle larvae (*Anisolemnia tetrastictas*: Dejean, Gibernau, Lauga, & Orivel, 2003) and jumping spiders, especially *Portia spp.* (e.g., Jackson & Pollard, 1996, and much subsequent work from this research group), in nest-site selection by Indian house crows (*Corvus splendens*: Yosef, Zduniak, Poliakov, & Fingerman, 2019), in the detection of water for drinking by barbastelle bats (*Barbastella barbastella*; Russo, Cistrone, & Jones, 2012), in the use of sponge tools by bottlenose dolphins (Krützen et al., 2005), and indeed in the variation of foraging behavior of Niligri langurs, as in the earliest use of the term “behavioral flexibility” that we have found (Poirier, 1969).

### Problem-solving capacity as behavioral flexibility

For many authors, the capacity for learning alone does not encompass what they mean by behavioral flexibility. A popular alternative, which has been used across a range of vertebrate taxa, is the capacity for learning the reversal of a task that has been well trained. Among those who have used reversal learning as an index of behavioral flexibility are Pintor, McGhee, Roche, and Bell (2014) studying Northern pike (*Esox lucius*); Petrazzini, Bisazza, Agrillo, and Lucon-Xiccato (2017) studying sex difference in the cognition of guppies (*Poecilia reticulata*); Szabo, Noble, Byrne, Tait, and Whiting (2018) studying tree skinks (*Egernia striolata*); Boogert, Monceau, and Lefebvre (2010) studying Zenaida doves (*Zenaida aurita*); Logan (2016b) studying great-tailed grackles (*Quiscalus mexicanus*); Gilbert-Norton, Shahan, and Shivik (2009) studying the effect of Skinnerian schedules of reinforcement on coyotes (*Canis latrans*); and Manrique and Call (2015) studying how great ape learning changes with age. This suggestion chimes with the popular view that a key feature of executive control in cognition is the capacity to inhibit a prepotent behavior, which is obviously implicated in reversal learning. A few authors have gone beyond that, and have considered that a better measure of behavioral flexibility would be the capacity for serial reversal learning, that is the capacity to “learn the rules” of reversal learning so that successive reversals are made more quickly. Examples include Liu, Day, Summers, and Burmeister (2016) in a study of the green and black poison dart frog *Dendrobates auratus* (they refer to serial reversal learning as requiring “advanced” behavioral flexibility); Bond, Kamil, and Balda (2007) in a comparative study involving three species of corvid; and Chow, Leaver, Wang and Lea (2015) studying Eastern gray squirrels.

In recent years, however, there has been an increasing tendency to use the term “behavioral flexibility” in particular connection with animal problem solving. Although any situation requiring learning can be described as solving a problem, “problem solving” typically refers to the spontaneous solution of physical problems, most often the extraction of food from inaccessible places. The places concerned could either be naturally occurring or experimentally contrived, but experimental situations – puzzle boxes – are easier to study, though they may be deployed in natural situations as well as in laboratories. Authors using the term “behavioral flexibility” either as a synonym for problem-solving ability or in an attempt to explain it, include Webster and Lefebvre (2001) in a comparative study of several species of birds in Barbados; Isden, Panayi, Dingle, and Madden (2013) studying spotted bowerbirds (*Ptilonorhynchus maculatus*); Mangalam and Singh (2013) examining urban bonnet macaques’ (*Macaca radiata*) strategies for extracting food from anthropogenic sources; Loukola, Perry, Coscos, and Chittka (2017) studying bumblebees (*Bombus terrestris*); and Chow, Lurz, and Lea (2018) comparing Eurasian red with Eastern gray squirrels (*Sciurus vulgaris* and *S. carolinensis*). Griffin and Guez (2014) reviewed the relation between experimental studies of problem solving and the emergence of innovative behaviors in the wild, invoking behavioral flexibility as a linking mechanism.

### Brain size, behavioral flexibility, and innovation

This tendency to identify behavioral flexibility with problem-solving ability is seen most prominently in attempts to make large-scale comparative assessments of cognitive differences between taxa, and to relate them to possible causes and consequences. The majority of these papers have come from Lefebvre and his colleagues. They have used published anecdotal reports of novel foraging methods in birds as an index of the species’ capacity for problem solving and hence of its behavioral flexibility, and have then examined how this measure correlates with residual forebrain size. Residual forebrain size is the excess of forebrain mass over what would be predicted from the correlation across a wide range of species between brain and body mass (cf. Jerison, 1985). Lefebvre, Whittle, Lascaris, and Finkelstein (1997) first demonstrated such a correlation between innovation reports and residual brain size across 17 different orders of birds, using data from North America and the British Isles, and Lefebvre, Gaxiola, Dawson, Timmermans, Rosza, and Kabai (1998) reported a similar correlation across orders and parvorders of Australasian birds. Similar correlations have been reported in other taxa, for example across species in the mammalian orders Primates (Reader & Laland, 2002) and Carnivora (Benson-Amram, Dantzer, Stricker, Swanson, & Holekamp, 2016). The basic analysis has also been refined. For example by Nicolakakis and Lefebvre (2000) using an enlarged sample of northern European birds, while Overington, Morand- Ferron, Boogert, and Lefebvre (2009) showed that the variety of innovations recorded was more important than the mere number of innovations. There have also been attempts to refine the areas of the brain responsible for the correlation: in Reader and Laland’s analysis, they focused on neocortex size, while Timmermans, Lefebvre, Boire, and Basu (2000) returned to the dataset used by Lefebvre et al. (1997), and showed that the key brain area in birds was the hyperstriatum. Because of the reliability of this correlation, residual brain size is sometimes used as a proxy for observed behavioral flexibility in comparative studies, as in the analysis carried through by Sol, Szekely, Liker, and Lefebvre (2007).

### The consequences of behavioral flexibility

There is a potential feedback relationship between behavioral flexibility and brain size: The greater the adaptive advantages of behavioral flexibility, the greater the selective pressure to increase the size of the brain. However, within the life of an individual organism, species brain size can reasonably be taken as a precursor of behavioral flexibility, rather than a consequence – although engaging in flexible behavior might cause brain growth, and has been claimed to induce desirable changes in brain activity (e.g., Belleville et al., 2011). But what might the consequences of higher behavioral flexibility be? Several surveys have suggested that, between taxa, higher behavioral flexibility contributes to fitness. Nicolakakis, Sol, and Lefebvre (2003) showed that, across the parvorders of birds worldwide, those with higher recorded innovation rates (hence, behavioral flexibility) tended to include more species, suggesting that evolution (and in particular, speciation) is speeded up in such taxonomic groups. Sol et al. (2007) extended this result by showing that, across both populations and taxonomic families of birds, higher residual brain mass is associated with lower adult mortality per year, even taking into account factors such as body mass and social structure. Within the parrots, Schuck-Paim, Alonso, and Ottoni (2008) showed that those with greater residual brain size (implying greater behavioral flexibility) tended to live in a wider range of climate types. A more ambiguous result was found by Sol, Lefebvre, and Rodriguez-Teijeiro (2005): They showed that migratory species of passerine bird tended to have lower residual brain size than related non-migrant species. Sol et al. interpret this result as implying that sedentary species have to rely on innovative feeding techniques to cope with winter conditions in the temperate zone, though it is not obvious why a change of seasons would impose more demands on foraging technique than a change of continents.

All these studies compare averages for species or higher taxonomic groups with one another. Within species, evidence that behavioral flexibility is correlated with fitness has been harder to find, regardless of how behavioral flexibility is measured. Within species, not all evidence supports the idea that flexibility enhances fitness: In their studies of spotted bowerbirds, Isden et al. (2013) used a battery of six cognitive tests including reversal learning, but could not derive any measure that would predict breeding success. Huebner, Fichtel, and Kappeler (2018) found a similar result in gray mouse lemurs (*Microcebus murinus*). And Madden, Langley, Whiteside, Beardsworth, and Van Horik (2018) found that speed of reversal learning in pheasant chicks (*Phasianus colchicus*) was negatively correlated with their survival as adults.

There is one particular context, however, in which many authors have associated greater success with higher behavioral flexibility, and that is in adaptation to anthropogenic environmental change and in particular to urbanization. The spread of urban development commonly results in a great loss of biological diversity in the wildlife of an area, but this may be accompanied by an increase in wildlife biomass; a few species do very well in the urban environment (e.g., Chace & Walsh, 2006). This may be due to dominance of resources by a few invasive species (Shochat et al., 2010). Both success in the urban environment and success in novel environments generally, and hence invasion success, have been repeatedly linked with behavioral flexibility (e.g., Griffin & Diquelou, 2015, for the case of the invasive Indian myna in Australia). There is an obvious logical link, in that to succeed in any new environment, and in particular in the urban environment, an animal has to be able to vary its behavior, but there is also substantial empirical evidence of a link, as demonstrated in the reviews by Sih (2013), Lowry, Lill, and Wong (2013) and Barrett, Stanton, and Benson-Amram (2019).

### So what does behavioral flexibility mean?

We have seen that across the past half-century “behavioral flexibility” has been used to mean everything from an absence of instinctual predetermination of behavior, through to relative performance under quite specific kinds of cognitive challenge. There is no simple historical progression between these different meanings, but the trend is towards using “behavioral flexibility” more specifically where learning or problem solving are demonstrably involved, with the term “phenotypic flexibility” being used where adaptive variation has an unknown origin, and might be due in part to genetic differences between individuals and populations.

Unsurprisingly, these different definitions do not cohere, empirically speaking. There are manipulations that leave problem solving unimpaired but damage learning of the reversal of the same problem (e.g., early social deprivation in rats; M. J. Morgan, 1973). Within species individual differences in simple associative learning speed are not reliably correlated with speed of solving more complex problems (e.g., in great-tailed grackles; Logan 2016a). Where a number of different cognitive tests are given to the same animals, correlations between their performance levels tend to be low (e.g., Isden et al., 2013). Specifically, the two capacities that have been most often associated with behavioral flexibility, reversal learning and innovative problem solving, do not necessarily correlate across individuals, as for example in the study of common mynas (*Sturnus tristis*) in Australia by Griffin, Guez, Lermite, and Patience (2013).

Inevitably, these conceptual and empirical inconsistences have resulted in some confusion (Audet & Lefebvre, 2017). One possible response would be to advise against using the term “behavioral flexibility” at all. However, we want to argue for a different approach: to give it a more precise meaning. The remainder of this paper explores how that might be done.

## A model of problem-solving incorporating flexibility

We present here a simple formal model of problem-solving, which includes a parameter to represent behavioral flexibility. Our model therefore focuses on behavioral flexibility in the sense of changes of behavior within an individual, rather than variations in behavior that can be attributed to stable differences between individuals, perhaps of genetic origin. On the other hand, the model is open to the possibility that the flexibility parameter is itself a stable difference between individuals, which might account for stable individual differences in problem-solving performance – where they exist (see Cauchoix et al., 2018).

Inevitably, such a model will not be a full representation of problem solving, and no parameter of any model will capture everything that has been meant by flexibility in the past. On the other hand, by constructing such a formal model, we force ourselves to be explicit about the roles that behavioral flexibility can play, and what is (and what is not) covered by our own emerging definition. We can also hope to make some counterintuitive or simply unexpected predictions about the effects of behavioral flexibility, predictions that could be tested in future experiments.

### The situation to be modeled

**Fig. 1:**
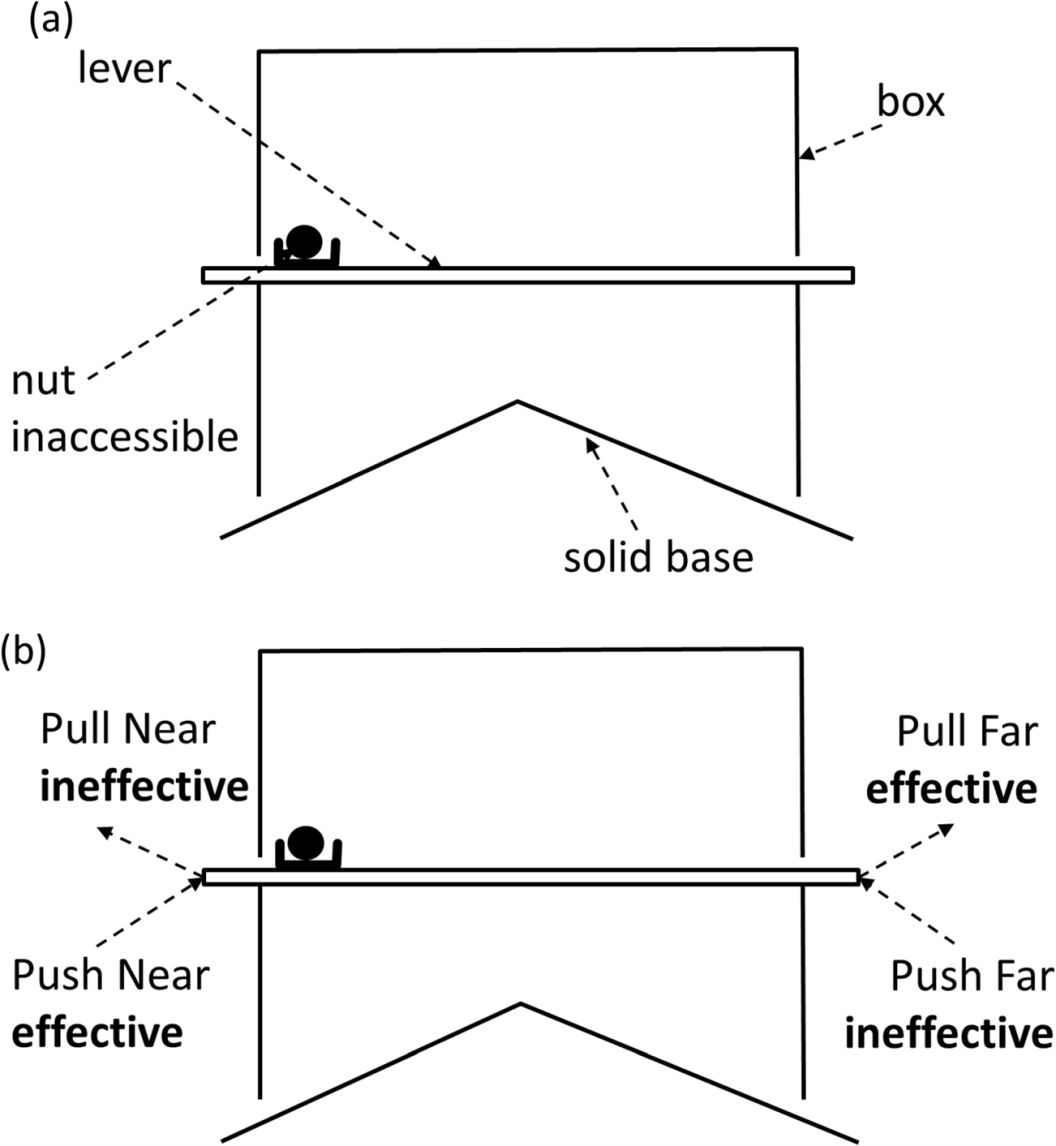
(**a**) Simplified diagram (not to scale) of the apparatus used by Chow et al. (2016). In practice there were ten levers, five of which were baited with nuts and five were unbaited. (**b**) Four possible responses a squirrel could make in an attempt to retrieve a nut

The puzzle situation that inspired this model has been used in a series of experiments on problem-solving in squirrels (Chow, Lea, & Leaver 2016; Chow et al., 2017; Chow, Lurz, & Lea 2018; Chow et al., 2019). The basic design is fully described by Chow et al. (2016), though later experiments involved some variations. It consists of a transparent, roughly cubical box supported on short legs above a solid pyramidal base. Slotted across the box are several levers, each bearing a container, which may (or may not) contain a nut. The squirrel’s task is to dislodge the levers that contain nuts; the nut will then fall to the base, and roll out of the apparatus so the squirrel can reach it. Figure 1a is a simplified diagram of the apparatus, with only a single lever represented. An important feature of the puzzle is that the nut container is at one end of the lever, and blocks the movement of the lever out of the box. Figure 1b shows four possible responses the squirrel might make to the lever: pulling it or pushing it, from either the end nearer the nut container (“near end”), or the end further from the nut container (“far end”). Of course, there are other possible responses as well.

This situation incorporates a number of features that we believe need to be included in any model of problem solving:

There are multiple possible responses that an animal can make.

Some responses are effective in solving the puzzle and thus securing the reward, others are not. Note, however, that the situation does not include any element of “shaping,” in which responses that are not fully effective are rewarded early in the animal’s exposure to the situation in order to encourage the emergence of more effective responses.

Some of the possible responses are more likely to be made than others when the animal first encounters the apparatus: they have a higher “operant level,” in the terms used by the early Skinnerians (e.g., Schoenfeld, Antonitis, & Bersh, 1950).

Independent of their operant level, with a given reward (in this case food), some responses will be learned more quickly than others: in the terms used by Seligman (1970), they have a higher level of preparedness.

### Basic principles of the model and their implementation

The key principle of our model is that not all learning is necessarily revealed in performance. The learning/performance distinction is one of the oldest ideas in animal learning theory, but historically it was invoked to account for the effects of motivational shifts. Here we use it to accommodate the effects of response competition: the fact that an animal has some tendency to emit several different responses, but only one of them can be emitted at any given moment. Accordingly, we draw a distinction between response probability, which is what we can actually observe, and response strength, an underlying, unobserved quantity that determines response probability in conjunction with the strengths of other, competing, responses. Because response strength is seen as an internal analog of response probability, we specify that it must have probability-like properties: all response strengths must lie between 0 and 1 (but never reach either of these limits), and at all times the sum of all response strengths is constant and equal to 1. Crucially, reward and non-reward are modeled as acting on response strengths, not directly on response probabilities.

To implement these principles, we need to specify the learning rule, to describe how response strengths are changed by reward and non-reward, and a response selection rule, to describe how a particular response is chosen given the strengths of all available responses. In our model, preparedness is modeled as a parameter of the learning rule, while flexibility, our topic of primary interest, is modeled as a parameter of the response selection rule. Thus, our model is implementing a hypothesis that behavioral flexibility is a matter of response selection rather than learning, though of course learning is still concerned indirectly, since it is learning that determines response strength. We note in passing that our hypothesis that flexibility is concerned with response selection opens the door to a possible distinction between behavioral and cognitive flexibility, a topic we have not broached in the present paper.

#### The learning rule

The choice of a particular learning rule is not crucial to our modeling, and we suspect that many different rules would give similar results. In the simulations that follow, we have used a simple linear rule:

Let *s*_i_(*t*) be the strength of the *i*th response at time *t*

Then whenever the *i*th response is emitted, *s*_i_(*t*) is changed to *s*_i_(*t*+1) where

1a $$ {s}_{\mathrm{i}}\left(t+1\right)=\left(1+{a}_{\mathrm{i}}\right){s}_{\mathrm{i}}(t)\ \mathrm{if}\ \mathrm{the}\ \mathrm{response}\ \mathrm{was}\ \mathrm{rewarded} $$

1b $$ {s}_{\mathrm{i}}\left(t+1\right)=\left(1-{b}_{\mathrm{i}}\right){s}_{\mathrm{i}}(t)\ \mathrm{if}\ \mathrm{the}\ \mathrm{response}\ \mathrm{was}\ \mathrm{not}\ \mathrm{rewarded} $$

Following these calculations, the *s*_i_ values are renormalized so that ∑*s*_i_ = 1. This has the consequence that, if the *i*th response is rewarded, the strengths of all other responses fall, while if *i*th response is emitted but not rewarded, the strengths of all other responses rise. Note that the application of these equations, and the subsequent normalization, will never result in an *s* value reaching 0 or 1.

The values *a*_i_ and *b*_i_ are response-specific parameters (though for simplicity they can be set equal across all responses). *a*_i_ represents the preparedness of response *i*: the higher *a*_i_, the easier it is to learn that response (for the given problem and reward type). Conversely, *b*_i_ represents the ease of extinction of response *i*. For any response, the relation between these two parameters is of particular significance: if *a*_i_ > *b*_i_, response *i* is resistant to extinction or perseverative – more is learned when it is successful than when it fails. The high resistance to extinction that can be seen after even a single reinforcement of a response (see, e.g., Skinner, 1938, Fig. 15) suggests that if this learning rule is to be used, *b* values might need to be set lower than corresponding *a* values.

#### The response-selection rule

**Fig. 2:**
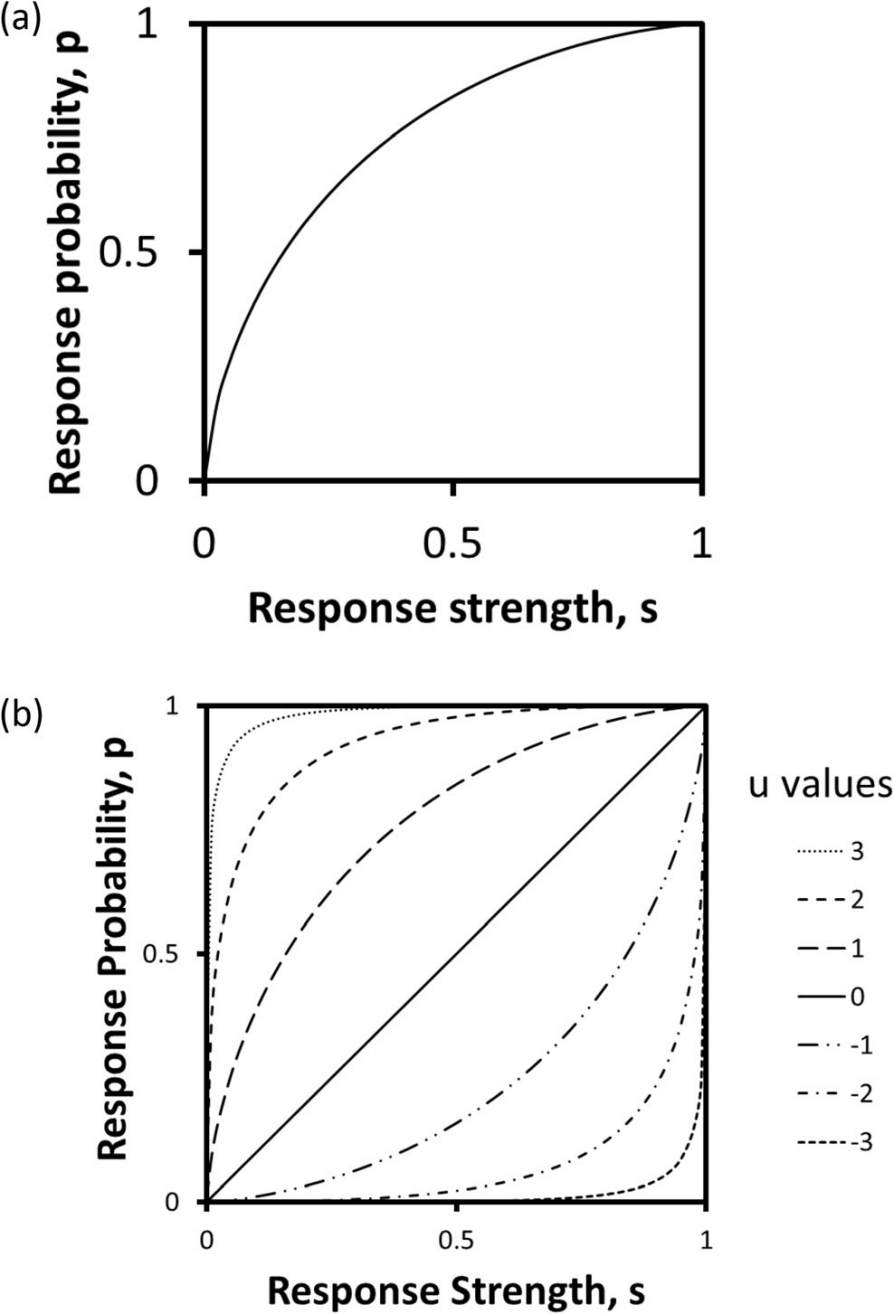
(**a**) The function used in the simulations to transform response strength, the quantity acted on by reward and non-reward, into response probability. The function is defined by Equation 3. (**b**) The effect of varying the parameter *u* in the function

The heart of our model is the response-selection rule. Formally, we can say that the probability of the *i*th response, *p*_i_, is determined by a transformation function *f*_u_

2 $$ {p}_{\mathrm{i}}={f}_{\mathrm{u}}\left({s}_{\mathrm{i}}\right) $$

where *u* is a parameter, intended to capture variations in behavioral flexibility; how it might do that is explored below. First, however, we need to specify some essential properties of the function *f*. From first principles, we can state: that *f* should be uniformly increasing in *s*, so that increasing response strength always results in increasing response probability; that as *s* approaches 0, *p* should correspondingly approach 0, so that a response that has negligible strength has a negligible chance of occurring; and that that as *s* approaches 1, *p* should correspondingly approach 1, so that a response that is the only one with any non-negligible strength is virtually certain to occur. Figure 2a shows one function that has these properties. It is certainly not the only candidate, but it is a convenient example. It is most easily described by a pair of equations:

3a $$ \upbeta ={N}^{-1}\left(1-{s}_{\mathrm{i}},0,1\right) $$

3b $$ {p}_{\mathrm{i}}=1-N\left(\upbeta, u,1\right) $$

where *N*(*x,m,σ*) is the integral from 0 to *x* of the normal distribution with mean *m* and standard deviation *σ,* and *N*^-1^(*y,m,σ*) is its inverse. This function is familiar as the receiver operating characteristic (ROC) used in the simplest form of psychophysical signal detection theory (Tanner & Swets, 1954), but our present use of it has no connection with signal detection. It is important to note that, because the *s*_i_ values are normalized so that they sum to one, it will also be necessary to normalize the *p*_i_ values following the application of Eq. 3, so that the property required of all probabilities, that ∑*p*_i_ = 1, is preserved.

The purpose of the parameter *u* is to represent behavioral flexibility, by changing the way in which response strengths are transformed into response probabilities. Figure 2b shows how the function *f* changes as a result of changes in *u*. With a zero value for *u*, we have a neutral situation, in which response strengths determine response probabilities directly, without transformation. With positive values of *u*, *p* values increase more rapidly than *s* values when *s* is low, and more slowly when *s* is high. This will lead to rapid initial learning following early rewards, but slower convergence on a single solution. Negative values of *u* lead to the converse case, where *p* values increase less rapidly than *s* values when *s* is low, and more rapidly when *s* is high. This should lead to more exploratory responding in the early stages of learning, but more rapid convergence once one response acquires dominant strength. Curiously, it is not immediately obvious which of these cases corresponds to greater behavioral flexibility, or would be more adaptive. We therefore postpone further interpretation of the parameter *u* until we have reported the results of simulations in which it is varied.

## Testing the model by simulation

Having defined the model, we then proceeded to test it by simulation. The simulation code was written in Pascal within the Embarcadero Delphi system, version XE2. The complete source code, and an executable file for the Windows 64-bit operating system, are available in an open repository at https://osf.io/uqdn3/.

### Initial simulations

We first tested the model’s properties by simulating a highly artificial situation. In these simulations, we set initial values and parameters as follows:

The repertoire size (the total number of responses considered) was set to 10. We gave all responses the same initial operant level, so all *s*_i_ values were initially set to 0.1; it follows that all *p*_i_ values also started at 0.1. There was no differential preparedness, so all *a*_i_ values were set to a constant value, *a*, of 0.1. Learning and extinction were regarded as symmetrical, so all *b*_i_ values were also set to 0.1. Only a single response was effective at any one time. In this and all our subsequent tests, we simulated the performance of the model by using random number generation to select a response according to the current set of *p*_i_ values.

We carried out 1,000 simulations of problem solving with these parameters and initial values, continuing training until the successful response was made nine times in ten successive responses; use of this criterion means that, regardless of the response strengths, the probability of the correct response should have reached about the same value, 0.9, in all situations. Once this criterion had been reached, we then changed which response was effective, but without changing the *s*_i_ values, so the simulated subject was faced with a shift or reversal situation.

Table 1 shows the results of these simulations, with three different values of *u* (-1, 0 and 1). It can be seen that changing the parameter *u* had a marked effect on the course of learning. With negative *u*, criterion was reached faster, both in initial learning and in reversal, but the correct response did not acquire such dominant strength. This will presumably have contributed to the more rapid learning of the shift. It is notable that, even after criterion has been reached on the shifted correct response, the initially correct response still has somewhat greater strength than the responses that were never rewarded. We confirmed that these trends were general by simulating with several other positive and negative values of *u*.

**Table 1:** Mean results of 1,000 simulations, in which each of ten responses was assigned the same operant level and preparedness. There was a single effective response in each phase of the simulation, and the model was trained until this response was made as nine out of ten successive responses. In the reversal phase, the effective response was changed, and the simulation started with the response strengths reached at the end of acquisition. Response strengths for the effective response at each phase are shown in bold

Phase of simulation	*u*	Mean responses to criterion	Response strengths (*s* values) at criterion
Acquisition (effective response no. 1)	-1	66.0	**.638**, .046, .046, .045, .042, .042, .048, .046, .046, .041
	0	95.3	**.818**, .023, .027, .023, .022, .029, .029, .027, .022, .029
	1	167.4	**.978**, .006, .005, .001, .008, .008, .003, .007, .005, .004
Reversal (effective response no. 2)	-1	85.6	.061**, .622**, .047, .032, .032, .034, .046, .039, .041, .035
	0	130.3	.032, .**821**, .015, .019, .018, .012, .015, .016, .013, .018
	1	193.3	.007**, .976**, .005, .007, .004, .003, .003, .001, .007, .005

On the basis of these results, we might suggest that negative *u* corresponds to greater behavioral flexibility, since it leads to more rapid learning both initially and following a change in reward contingencies. However, we wanted to test the model in a somewhat more realistic situation, making use of the parametric flexibility we had built into it.

### Simulations of an idealized version of the Chow et al. (2016) task

Accordingly, we selected parameters that plausibly described the problem-solving task used by Chow et al. (2016, 2017, 2018, 2019) involving the apparatus shown in Fig. 1. We used a slightly idealized version of the task, based on how we initially expected squirrels to respond to it, rather than on the way they actually responded, because that allowed us to pit the effects of operant level and preparedness against each other, whereas in practice Chow et al. (2016) found the same responses were favored by both operant level and preparedness. The initial values and parameters we used were as follows. In the light of the initial simulations reported above, we set repertoire size at 8, since that seemed sufficient to show the effects of experience on non-rewarded responses. We focused on four of these responses, to correspond to the Push Near, Pull Near, Push Far, and Pull Far responses shown in Fig. 1. By the design of the apparatus, only Push Near and Pull Far would be effective. However, we assumed that the two Pull responses would have higher operant levels than the two Push responses, since they involved moving the nut towards the squirrel rather than away from it. Finally, we assumed that Near responses would be more prepared than Far responses, since Far responses require a detour, and it has been known at least since Köhler (1925, chapter 1) that problem solutions involving detour are generally slow to emerge.

Our assumptions do not fully correspond to what Chow et al. (2016) observed. As we expected, they did indeed observe that Pull responses were learned less readily than Push responses. However, they also found that their operant levels were lower than those of Push responses, contrary to what we expected. Accordingly, we cannot be sure that the rapid learning of Push was due to lower preparedness. There is no interest in simulating a situation where both operant level and preparedness favor the same responses, since in that case the effects of the two factors cannot be disentangled. It is in this sense that we are simulating an idealized version of the Chow et al. experiment.

In the light of these considerations we set initial values and parameters as follows:

- Push Near: initial *s* = 0.05 (low operant level), *a* = 0.2 (high preparedness)
- Push Far: initial *s* = 0.05 (low operant level), *a* = 0.05 (low preparedness)
- Pull Near: initial *s* = 0.2 (high operant level), *a* = 0.2 (high preparedness)
- Pull Far: initial *s* = 0.2 (high operant level), *a* = 0.05 (low preparedness)

The remaining four responses were given neutral values of *s* and *a*, 0.1 in both cases. Recognizing that symmetry between acquisition and extinction is unrealistic, we set all *b* values to half the corresponding *a* values.

Following the procedure used by Chow et al. (2016), we simulated training up to the point of 60 successful responses. Relative success at learning was therefore measured by the total number of responses emitted before this criterion was reached. We varied the parameter *u* in the same way as in the initial simulations reported above, and ran 10,000 simulations with each *u* value.

**Fig. 3:**
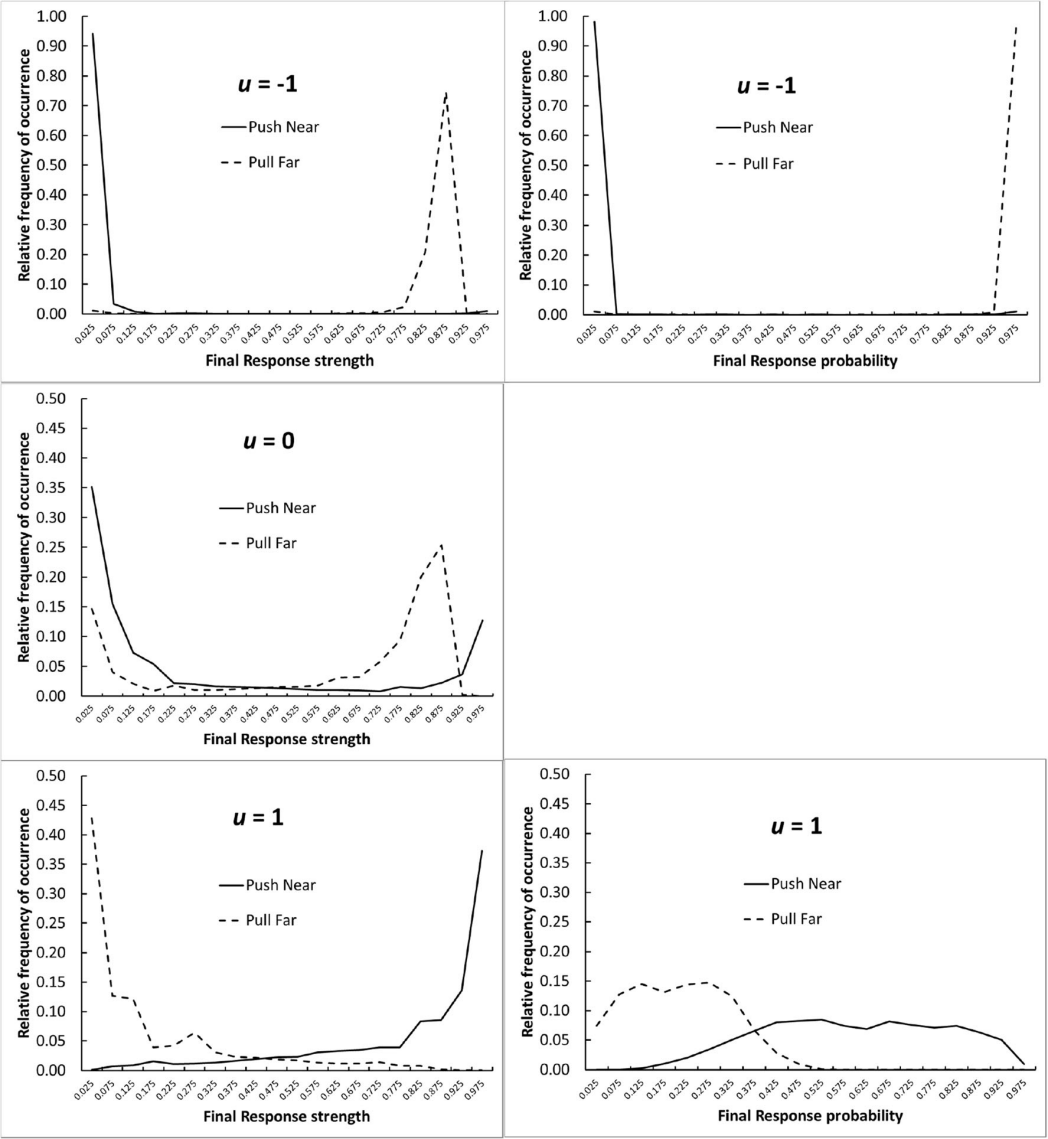
Histograms of final response strengths and probabilities obtained in the simulations of an idealized version of the experiment of Chow et al. (2016). Note that for the case of *u* = 0, response probabilities are identical to response strengths. Note also that a different vertical scale is used in the case of *u* = -1

Table 2 shows the results of these simulations. As in the initial simulations, negative *u* values led to faster learning, in that fewer total responses were made in the course of making 60 effective responses. However, this was not the only effect of varying *u*. With *u* set to -1, the simulations almost always locked onto the Pull Far response (high operant level, low preparedness). But with *u* set to +1, they most often locked on to the alternative effective response, Push Near, with low operant level but high preparedness. With the neutral value of *u*, response probabilities to the two effective responses were intermediate, with both occurring with substantial probability. These intermediate mean values for response strengths and probabilities are ambiguous, in that an intermediate value could arise in two ways. It could be that the final *s* and *p* values are indeed intermediate, or it could be that they are always take extreme values, close to zero or one, but that sometimes they go to one extreme and sometimes to the other. To distinguish these two possibilities, we examined the results of individual simulations. Figure 3 shows histograms of final *s* and *p* values for the two effective responses, for the three different values of *u* tested.

**Table 2:** Mean results of 10,000 simulations of an idealized version of the problem used by Chow et al. (2016). The first four responses for which data are shown in the right-hand columns correspond to the Push Near, Push Far, Pull Near, and Pull Far responses shown in Fig. 1b; these responses differed in operant level (Pull higher than Push) and preparedness (Near higher than Far). The remaining four responses had equal, intermediate levels of these quantities. Response strengths and probabilities for the effective response at each phase are shown in bold

u	Mean responses to 60 successes	Mean response strengths after 60 successes	Response probabilities calculated from final mean response strengths
-1	83.3	**.035**, .012, .025, .**847,** .024, .026, .028, .027	**.004**, .003, .006, .**988,** .005, .003, .009, .001
0	103.9	**.315**, .012, .014, .**595**, .017, .018, .014, .019	**.318**, .013, .012, .**595**, .011, .016, .013, .012
1	130.5	**.805**, .002, .003, .**154**, .006, .003, .001, .007	**.521**, .028, .039, .**266**, .034, .036, .033, .037

The histograms shown in Fig. 3 confirm that, with *u* set to -1, the simulation almost always locks on to the Pull Far response. With *u* set to zero, it tends to lock on to one of the effective responses or the other, though it is unpredictable which will be preferred, and there is a modest frequency of cases where both occur. With *u* of 1, the majority tendency is for the simulation to lock onto the Push Near response, but there is a higher frequency of mixed outcomes; and the final response probabilities are strikingly less extreme than the final response strengths.

### Conclusions from the simulations

Does our parameter *u* capture behavioral flexibility, as we hoped? We argue that it does, up to a point, with negative *u* corresponding to higher behavioral flexibility than positive *u*. In all situations we have tested, negative *u* leads to faster learning; and when we set operant level against preparedness, in simulating the experiments of Chow et al. (2016), a negative *u* value enabled what was learned to be controlled by operant level (which would be a commonsense expectation in any situation) rather than being captured by preparedness. In both these senses, therefore, negative *u* is associated with freedom from instinctual constraints – and as we saw in the first part of the present paper, that was the original meaning of behavioral flexibility, and still perhaps represents its core meaning. Of course, operant levels might themselves result from the animal’s instinctual repertoire, but they need not: so far as our model is concerned, they are simply the outcome of the response strengths with which the animal approaches the situation, and these will be the result of all its previous experience as well as any innate tendencies.

A further way in which negative *u* values can be seen to represent greater behavioral flexibility can be seen by reference to Table 1. In our simple, initial simulations, we trained to a stringent criterion (9 effective responses out of 10), and this required a high probability of emitting the effective response. With positive or zero *u*, the resulting response strengths were also close to one, meaning that all other responses had very little strength. But with negative *u*, the strength of the preferred response was not entirely dominant, meaning that when the situation changes, the simulation is able to change its behavior more quickly. This point emphasizes the significance of the key assumption in all our simulations, that not everything that is learned is immediately expressed in behavior. It may well be that it is an animal’s currently unexpressed learning that enables it to behave flexibly.

However, *u* clearly does not capture everything that has been meant by behavioral flexibility. In particular, we have not found that manipulating *u* can dissociate learning speeds in situations that have been thought to require different degrees of flexibility, for example reversal learning compared with initial learning.

As Audet and Lefebvre (2017) have pointed out, the different ways in which different authors have used the term “behavioral flexibility,” and the different ways in which they have sought to measure it, can only lead to conceptual confusion. Ultimately it is an empirical matter which of the behavioral traits that might be thought to represent flexibility will turn out to be correlated across individuals. However, theoretical analysis and simulation can help by demonstrating that single points of variation, such as manipulation of our parameter *u*, may have multiple effects all of which are plausible signs of flexibility – but, as in our results, may fail to produce other effects that have also been thought to be such signs.

### Limitations of the model

The present model is, of course, not a complete representation of the kinds of learning that can take place in problem-solving situations. Three well-known phenomena, in particular, are not represented within it:

1. Inhibitory learning. In our model, reward increases response strength, and non-reward decreases it. Although the rates at which these processes can be made to differ (by making our *a* and *b* parameters unequal), there is no representation of history. But we have known since the work of Pavlov (1927, Lecture IV) that extinguishing a response does not return it to the state before it was ever rewarded. Although our model does separate response probability from the underlying response strength, it is not clear that it could ever predict phenomena such as spontaneous recovery; to do that one would have to introduce separate values for excitatory and inhibitory response strength, as Pavlov did. That would be possible within our model, but it would introduce substantial extra complexity, which might make it difficult to get clear and consistent results from simulations.
2. Perseveration. In Thorndike’s (1913) initial formulation of the laws of learning, he included a Law of Exercise, which simply stated that, regardless of reward, the more often a response had occurred, the more often it is likely to occur in the future. Although Thorndike diluted this law in his later formulations (e.g., Thorndike, 1940), it remains true that animals are commonly observed to perseverate on responses even when there is no reward for doing so (e.g., M.J. Morgan, 1974), and our model does not allow for any such tendency – though it might be possible to do so with a relatively minor modification.
3. Generalization. The basis of shaping, whether it occurs accidentally as in Lloyd Morgan’s (C.L. Morgan, 1894, pp. 291-294) observations of problem solving in his dog Tony, or deliberately as noted by Skinner (1938, pp. 339-340) and much standard laboratory practice that has followed from his work, is that rewarding one response will make some other responses more likely. Such response generalization could be included in our model, though it would bring an unwelcome increase in the number of parameters to be specified: a matrix of generalization coefficients linking each response to all others would be required.

Although all of these processes are basic to animal learning and problem solving, and we could certainly add others to them, we believe that none of them is inherent to the concept of behavioral flexibility; so it is unlikely that a more complex model that included these processes would produce very different results from those we have already seen.

## General conclusions

Both from our historical exploration of the literature that has used the term “behavioral flexibility” and from our attempts to represent it within a formal model of problem solving, a core meaning for this protean term emerges. It refers to a quality or trait that frees an animal from the constraints of instinct, and allows it to adapt efficiently to variation in the environment. Such a trait might lead to variations between populations of a species that are faced with different environmental demands; it may also lead to different individuals finding different solutions to problems that all members of their species face. We have not here considered flexibility as a personality dimension, but it seems highly likely that it will vary between individuals within a species, just as it self-evidently varies between species.

That core meaning, however, is not sufficient to specify in all cases whether a given usage of the term “behavioral flexibility” is appropriate or not. Only empirical work will determine whether all tendencies that fit that broad definition in fact co-vary, either between or within species; and only theoretical work will determine whether tendencies that do co-vary can be attributed to variations in a single property or mechanism. The present simulations have shown that some, but not all, of the effects that have been ascribed to variations in behavioral flexibility can be modeled by manipulating a single parameter in a simple model of problem solving. We believe that this is a promising direction of research, and could usefully be extended.

We do not suppose that the function we have proposed to relate response probabilities to response strengths has any physical reality in any animal’s brain. What it does is express an idea: the recognition that what an animal does, at any point in time, does not completely reflect what it has learned – what it knows, if you will. In its most abstract form, therefore, what we are arguing is that the key to behavioral flexibility lies in the knowledge that an animal has, beyond what it is currently expressing in its behavior. It is not unreasonable to suppose that the wider and deeper that knowledge, the better equipped the animal will be to cope with new situations; in a word, the better it will be able to show flexibility.

### Open practices statement

No original data are reported in this paper. The source code of the simulation program used, and an executable file for the Windows 10 environment, are available in an open repository. Detailed results of the simulations summarized in the tables can be found in the same repository at https://osf.io/uqdn3/.

### Author notes

Pizza Chow is now at the Comparative Cognition Research Group, Max Planck Institute for Ornithology, Siewiesen, Germany. The authors declare that they have no conflict of interest.

Some of the material covered in this paper was presented by Stephen Lea in a Master Lecture at the meeting of the Comparative Cognition Society held in Melbourne, Florida, USA in April 2019. We are grateful for comments and suggestions made by colleagues present at that meeting.
